# Beyond acceptance: bounded use of AI and VR in orthopedic education

**DOI:** 10.3389/fmed.2026.1856528

**Published:** 2026-07-15

**Authors:** Xiaohong Lyu, Yiwei Zhao, Siyi Cai, Jianguo Zhang

**Affiliations:** 1 Department of Breast Surgery, Peking Union Medical College Hospital, Chinese Academy of Medical Sciences and Peking Union Medical College, Beijing, China; 2 Department of Orthopedics, Peking Union Medical College Hospital, Chinese Academy of Medical Sciences and Peking Union Medical College, Beijing, China

**Keywords:** artificial intelligence, medical education, medical students, orthopedic education, virtual reality

## Abstract

**Background:**

The educational roles of artificial intelligence (AI) and virtual reality (VR) in orthopedic training remain insufficiently defined. Rather than examining acceptance alone, we investigated how students delineate trust boundaries, role allocation, and implementation priorities for bounded, faculty-supervised use of AI/VR in orthopedic education.

**Methods:**

We conducted an exploratory cross-sectional online survey among Years 4–8 students in the eight-year program of clinical medicine at Peking Union Medical College using a study-specific questionnaire informed by AI- and simulation-enhanced medical education. Approximately 300 students were invited; 56 valid responses were analyzed (response proportion, 18.7%). The questionnaire assessed prior exposure to AI/VR, acceptance, perceived value, trust-related attitudes, application preferences, substitution boundaries, and risk perceptions.

**Results:**

Overall, 83.9% of respondents supported or strongly supported further AI/VR integration in orthopedic teaching. Acceptance showed the highest composite score (4.25 ± 0.73), followed by perceived value (4.22 ± 0.61), educational trust (4.02 ± 0.49), and risk perception (3.80 ± 0.53). Support for AI-assisted tools and VR-based simulation was similarly high (4.29 ± 0.73 vs. 4.32 ± 0.83, *p =* 0.694). The strongest endorsements were for continued in-person teacher guidance (4.59 ± 0.53) and teacher review of AI/VR content before formal use (4.52 ± 0.57), whereas trust in AI-generated recommendations for real patient decision-making was the lowest-rated item (3.11 ± 1.11). Students consistently favored augmentation over replacement: AI was preferred for knowledge summarization, reasoning support, and examination preparation, whereas VR was preferred for anatomy learning, fracture classification, surgical pathway visualization, and pre-training. Prior VR exposure was associated with greater implementation support (4.41 ± 0.51 vs. 3.85 ± 0.90, *p =* 0.014).

**Conclusion:**

Students supported AI/VR in orthopedic education, but within clear pedagogical and governance boundaries. Their responses suggest a preliminary, role-specific implementation model in which AI primarily enhances cognitive learning and VR primarily supports spatial-procedural training, under sustained faculty oversight. These perception-based findings should be tested in longitudinal or interventional curriculum studies.

## 1 Introduction

Artificial intelligence (AI) and virtual reality (VR) are increasingly reshaping medical education by expanding opportunities for adaptive tutoring, feedback, knowledge organization, simulation, and experiential learning (1–3). Recent studies suggest that these technologies can improve engagement, accessibility, and personalization, but also emphasize that educational value depends on careful curricular integration rather than technological novelty alone (4–6). At the same time, medical students’ attitudes toward AI remain conditional: although many perceive AI as useful for explanation, revision, and efficiency, concerns about inaccuracy, overreliance, bias, and weakened critical thinking persist, particularly when AI approaches clinically consequential decision-making (7–9).

These issues are especially relevant to orthopedic education. Orthopedic learning requires not only conceptual understanding, but also three-dimensional anatomical reasoning, fracture classification, procedural visualization, and preparation for hands-on performance (10–12). VR appears particularly suited to spatial and procedural training, whereas AI may be more useful for knowledge summarization, case-based reasoning support, and learning efficiency (13–15). However, the educational roles of AI and VR are unlikely to be identical, and their appropriate degree of autonomy within specialty teaching remains insufficiently defined.

Despite growing interest in digital technologies in medical education, comparatively few studies have examined how students conceptualize the joint use of AI and VR within a single specialty curriculum, especially with respect to trust boundaries, substitution boundaries, and scenario-specific application preferences in orthopedic education, particularly in the Chinese context (16–18). Because such perceptions directly influence implementation feasibility and curriculum design, we conducted an exploratory cross-sectional questionnaire study among students in the eight-year program of clinical medicine at Peking Union Medical College. We aimed to examine acceptance, perceived value, trust boundaries, preferred roles, application priorities, and risk perceptions related to AI/VR-assisted orthopedic education, and to generate preliminary implementation considerations for faculty-supervised specialty teaching. We hypothesized that students would show high overall support for AI/VR integration, but would favor augmentation over replacement and would express stronger trust in educational support functions than in autonomous clinical decision-making.

## 2 Methods

### 2.1 Study design and participants

This exploratory cross-sectional questionnaire study investigated perceptions of AI- and VR-assisted orthopedic education among students enrolled in the eight-year program of clinical medicine at Peking Union Medical College (PUMC), Beijing, China. The survey targeted students from Year 4 through 8, representing the main clinical-stage cohorts with potential exposure to orthopedic learning activities. Because the survey targeted Years 4–8 students, respondents were reported as Year 4, Years 5–6, and Years 7–8 in the final analysis. The questionnaire was distributed to approximately 300 eligible students across five grade levels through the Wenjuanxing online survey platform. We attempted to invite the full eligible cohort rather than sample a predefined subgroup; therefore, no *a priori* power calculation was performed. Participation was voluntary and anonymous. A total of 56 complete and valid responses were included in the final analysis, corresponding to a response proportion of 18.7%.

### 2.2 Questionnaire development and administration

The questionnaire was developed specifically for this study to assess students’ acceptance of AI/VR-assisted orthopedic education, perceived educational value, trust boundaries, application preferences, and risk perceptions. The original instrument comprised 53 items organized into eight sections: demographic characteristics, prior exposure to AI/VR tools, overall acceptance, perceived value, trust boundaries, substitution boundaries and application preferences, risk perception, and overall attitude/open-ended items. Most attitudinal items were measured on 5-point Likert scales ranging from 1 (strongly disagree) to 5 (strongly agree). Single-choice and multiple-choice items were used for demographic variables, prior exposure, preferred roles, teaching scenarios, and desired platform functions.

Item content was informed by the study objectives, prior literature on AI- and simulation-enhanced medical education, and discussion within the author team, which included clinicians and educators involved in orthopedic or medical teaching. Items were reviewed for face and content relevance before survey release and revised for clarity. The final questionnaire was implemented in Chinese on Wenjuanxing. An English translation of the questionnaire is provided as Supplementary Appendix 1. Because this was an exploratory single-center survey with 56 valid responses, formal Delphi validation, pilot psychometric testing, and exploratory factor analysis were not performed; construct validity therefore requires further evaluation in larger multi-center samples.

### 2.3 Measures and scoring

Four multi-item domains were prespecified according to the questionnaire structure: acceptance (Q14–Q21), perceived value (Q22–Q29), trust-related attitudes (Q30–Q37), and risk perception (Q45–Q50). Composite domain scores were calculated as the arithmetic mean of the corresponding item responses, with all items weighted equally. No items were reverse-coded. Higher scores indicated stronger endorsement of the statements within each domain. Overall support for further implementation of AI/VR in orthopedic teaching was assessed using Q51. For key Likert items, the proportion of respondents selecting ‘agree’ or ‘strongly agree’ was also calculated to enhance interpretability. The four domains were defined *a priori* to correspond to the study objectives: the acceptance domain captured students’ general willingness to adopt AI/VR in orthopedic teaching; the perceived value domain captured the educational benefit attributed to these tools across knowledge, reasoning, and spatial-procedural learning; the trust-related domain captured the degree to which students were willing to rely on AI/VR outputs and the point beyond which faculty validation was considered necessary; and the risk perception domain captured concerns about accuracy, over-reliance, ethics, and data governance. Individual items were assigned to a domain by author-team consensus based on their conceptual correspondence to that construct, and items addressing distinct constructs (substitution boundaries and application preferences) were analyzed separately rather than combined into these composites.

### 2.4 Statistical analysis

Data were exported from Wenjuanxing and analyzed using Python. Descriptive statistics are presented as number (percentage) for categorical variables and as mean ± standard deviation (SD) for Likert-derived continuous variables and composite scores. We summarized multi-item Likert domain scores as approximately continuous variables because they represented prespecified composite constructs, while also reporting agreement proportions for key single items. Internal consistency was assessed using Cronbach’s alpha. Paired Wilcoxon signed-rank tests were used for within-participant comparisons between conceptually related items. Mann–Whitney U tests were used for two-group comparisons of Likert-derived outcomes, and Fisher’s exact test was used for dichotomized support outcomes. For Wilcoxon and Mann–Whitney tests, effect sizes were reported as *r* = |Z|/sqrt(*N*). Associations between self-rated AI/VR familiarity and composite domain scores or overall support were examined using Spearman rank correlation coefficients. Subgroup analyses were exploratory, and no correction for multiple comparisons was applied. All tests were two-sided, and *p* < 0.05 was considered statistically significant. Complete-case analysis was performed, and no imputation for missing data was required because only valid questionnaires were included. We recognize that treating Likert-derived composite scores as approximately continuous remains methodologically debated, because such data are strictly ordinal and some authors recommend ordinal or rank-based approaches. To mitigate this concern, we relied on non-parametric tests for all between-group and within-participant comparisons and reported agreement proportions alongside composite means to preserve interpretability.

## 3 Results

### 3.1 Participant characteristics and prior exposure

A total of 56 valid questionnaires were included in the analysis. The sample comprised 30 female students (53.6%), 25 male students (44.6%), and one respondent who preferred not to disclose sex (1.8%). Most participants were in Years 5–6 (41.1%) or Year 4 (37.5%), whereas 21.4% were in Years 7–8. Thirty-five students (62.5%) had already received systematic orthopedic theory teaching, and 22 (39.3%) had participated in orthopedic observation, internship, or rotation activities. Notably, 34 students (60.7%) had never observed an orthopedic operation. With respect to digital learning exposure, 44 students (78.6%) reported using AI tools often or almost daily for medical study, whereas only 17 (30.4%) had prior exposure to VR/AR/simulation systems in medical learning (Table 1).

**Table 1:** Participant characteristics and prior learning exposure (*n* = 56).

Characteristic	*n*	%
Sex		
Male	25	44.6
Female	30	53.6
Prefer not to say	1	1.8
Training stage		
Year 4	21	37.5
Years 5–6	23	41.1
Years 7–8	12	21.4
Received systematic orthopedic theory teaching		
Yes	35	62.5
No	21	37.5
Participated in orthopedic observation/internship/rotation		
Yes	22	39.3
No	34	60.7
Observed orthopedic surgery		
Never	34	60.7
1–2 times	9	16.1
3–5 times	7	12.5
>5 times	6	10.7
Considering a surgical specialty in the future		
Yes	26	46.4
No	23	41.1
Uncertain	7	12.5
Considering an orthopedic specialty in the future		
Yes	1	1.8
No	44	78.6
Uncertain	11	19.6
Frequency of AI-assisted medical learning		
Never	1	1.8
Rarely	1	1.8
Occasionally	10	17.9
Often	28	50.0
Almost daily	16	28.6
Prior exposure to VR/AR/simulation in medical learning		
Yes	17	30.4
No	39	69.6

Data are presented as *n* (%) unless otherwise indicated.

### 3.2 Internal consistency and domain-level scores

**Figure 1:**
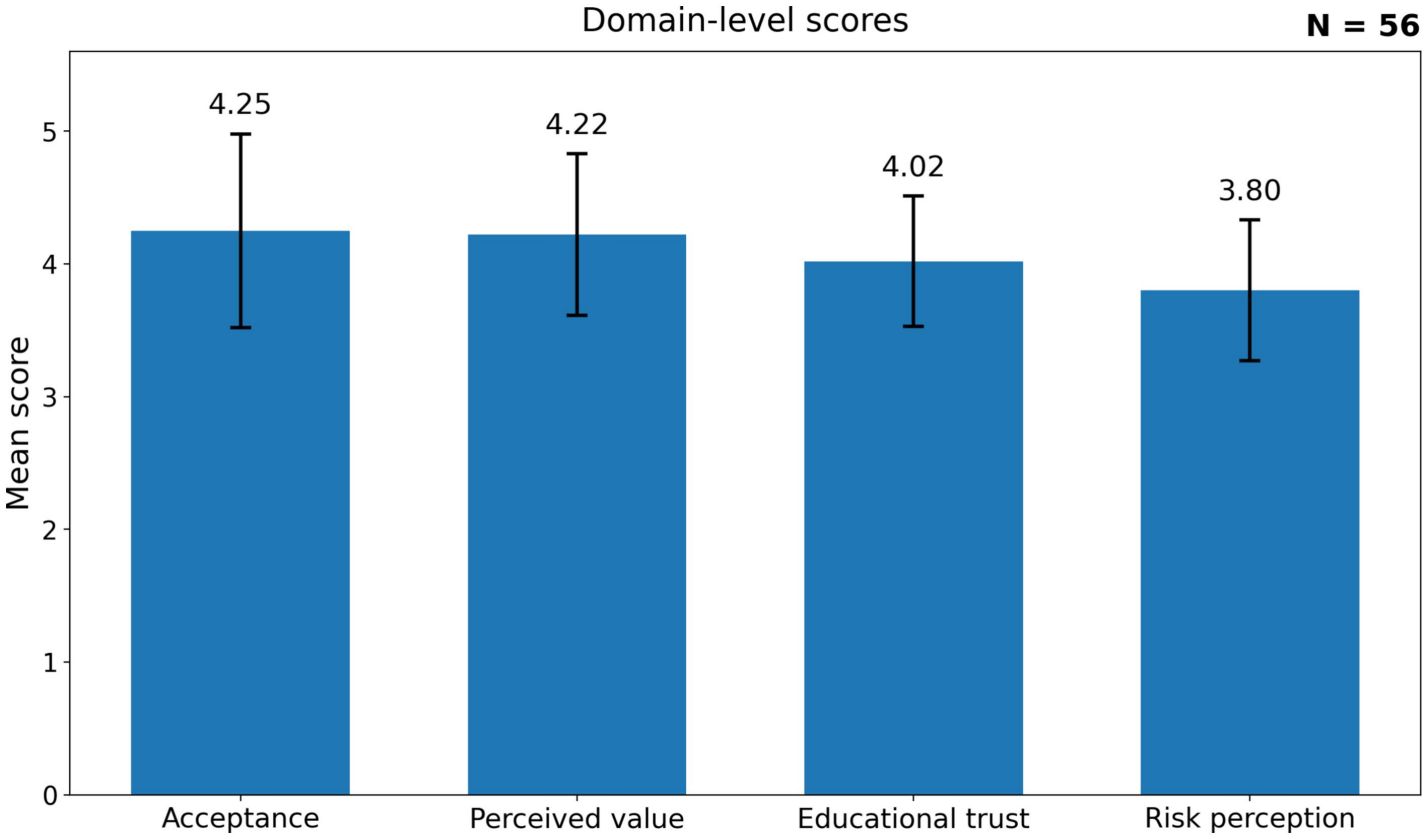
Domain-level scores showed high acceptance and perceived value of AI/VR-assisted orthopedic education, together with nontrivial risk awareness.

Internal consistency was high for the acceptance domain (Cronbach’s *α* = 0.952) and the perceived value domain (*α* = 0.902), acceptable for the educational trust domain (*α* = 0.805), and adequate for the risk perception domain (*α* = 0.710). The highest domain-level mean score was observed for acceptance (4.25 ± 0.73), followed by perceived value (4.22 ± 0.61), educational trust (4.02 ± 0.49), and risk perception (3.80 ± 0.53), suggesting strong endorsement of AI/VR-assisted orthopedic education together with persistent caution regarding implementation risks (Table 2; Figure 1).

**Table 2:** Psychometric performance and domain-level scores.

Domain	No. of items	Cronbach’s alpha	Mean ± SD
Acceptance	8	0.952	4.25 ± 0.73
Perceived value	8	0.902	4.22 ± 0.61
Educational trust	8	0.805	4.02 ± 0.49
Risk perception	6	0.710	3.80 ± 0.53

Domain scores were calculated as the arithmetic mean of Likert items scored from 1 to 5.

Composite mean scores for acceptance, perceived value, trust-related attitudes, and risk perception among 56 respondents on 5-point Likert scales. Each domain score was calculated as the equally weighted mean of prespecified items. Error bars indicate standard deviations. Higher scores indicate stronger endorsement of the corresponding construct.

### 3.3 Overall acceptance and support

Overall support for further implementation of AI/VR in the 8-year orthopedic curriculum was high: 47 of 56 students (83.9%) selected “support” or “strongly support”, whereas only two respondents (3.6%) selected “strongly do not support.” Students expressed similarly high endorsement for the introduction of AI-assisted learning tools (4.29 ± 0.73) and VR/immersive simulation training (4.32 ± 0.83), with no significant difference between the two modalities (Wilcoxon signed-rank test, *p* = 0.694; effect size *r* = 0.10). Among the acceptance items, the strongest endorsement was observed for the statement that AI/VR can serve as a useful supplement to traditional orthopedic teaching (4.43 ± 0.74; 94.6% agreeing or strongly agreeing) and that AI/VR can improve the intuitiveness and comprehensibility of orthopedic teaching (4.43 ± 0.85; 89.3% agreeing or strongly agreeing).

### 3.4 Perceived educational value and trust boundaries

Perceived educational value was particularly high for VR in spatially and procedurally complex learning tasks. The highest value-related items were “VR helps me engage in pre-training before formal clinical teaching” (4.34 ± 0.82; 87.5% agreement), “VR helps me understand fracture classification, internal fixation, and surgical pathways” (4.32 ± 0.74; 92.9% agreement), and “VR helps me understand skeletal, joint, and spatial anatomy” (4.27 ± 0.84; 87.5% agreement). AI also received favorable evaluations for supporting foundational knowledge acquisition and examination preparation, with mean scores above 4.0 across all three AI-related value items.

A clear trust boundary emerged when students distinguished between educational support and direct clinical decision-making. The two most strongly endorsed trust statements were that orthopedic teaching still requires in-person teacher guidance even if AI/VR performs well (4.59 ± 0.53; 98.2% agreement) and that AI/VR content should undergo teacher review before formal teaching (4.52 ± 0.57; 96.4% agreement). By contrast, trust in AI-generated orthopedic recommendations for real patient decision-making was the lowest-scoring item in the entire questionnaire (3.11 ± 1.11; 33.9% agreement). Trust in VR for procedural workflow demonstration was significantly higher than trust in AI-generated recommendations for real patient decision-making (4.29 ± 0.76 vs. 3.11 ± 1.11, *p* < 0.001; effect size *r* = 0.79), and the perceived necessity of teacher guidance was likewise significantly higher than trust in AI-generated recommendations for real patient decision-making (4.59 ± 0.53 vs. 3.11 ± 1.11, *p* < 0.001; effect size *r* = 0.87; Table 3).

**Table 3:** Boundary-defining items across the questionnaire: strongest endorsements, lowest trust, and salient implementation concerns.

Statement	Mean ± SD	Agree, *N* (%)
Even if AI/VR performs well, orthopedic teaching still requires in-person teacher guidance.	4.59 ± 0.53	55 (98.2)
AI/VR educational content should be formally taught only after teacher review.	4.52 ± 0.57	54 (96.4)
AI/VR can serve as a useful supplement to traditional orthopedic teaching.	4.43 ± 0.74	53 (94.6)
AI/VR can improve the intuitiveness and comprehensibility of orthopedic teaching.	4.43 ± 0.85	50 (89.3)
I trust AI-generated orthopedic clinical recommendations sufficiently for real patient decision-making.	3.11 ± 1.11	19 (33.9)
I worry that AI/VR may weaken authentic teacher–student interaction.	3.29 ± 1.04	25 (44.6)
I worry that AI/VR may lead students to overestimate their actual clinical competence.	3.46 ± 0.99	31 (55.4)
I trust AI explanations of foundational orthopedic knowledge.	3.71 ± 0.71	34 (60.7)

Agreement was defined as a Likert score of 4 or 5.

### 3.5 Preferred roles and application priorities

**Figure 2:**
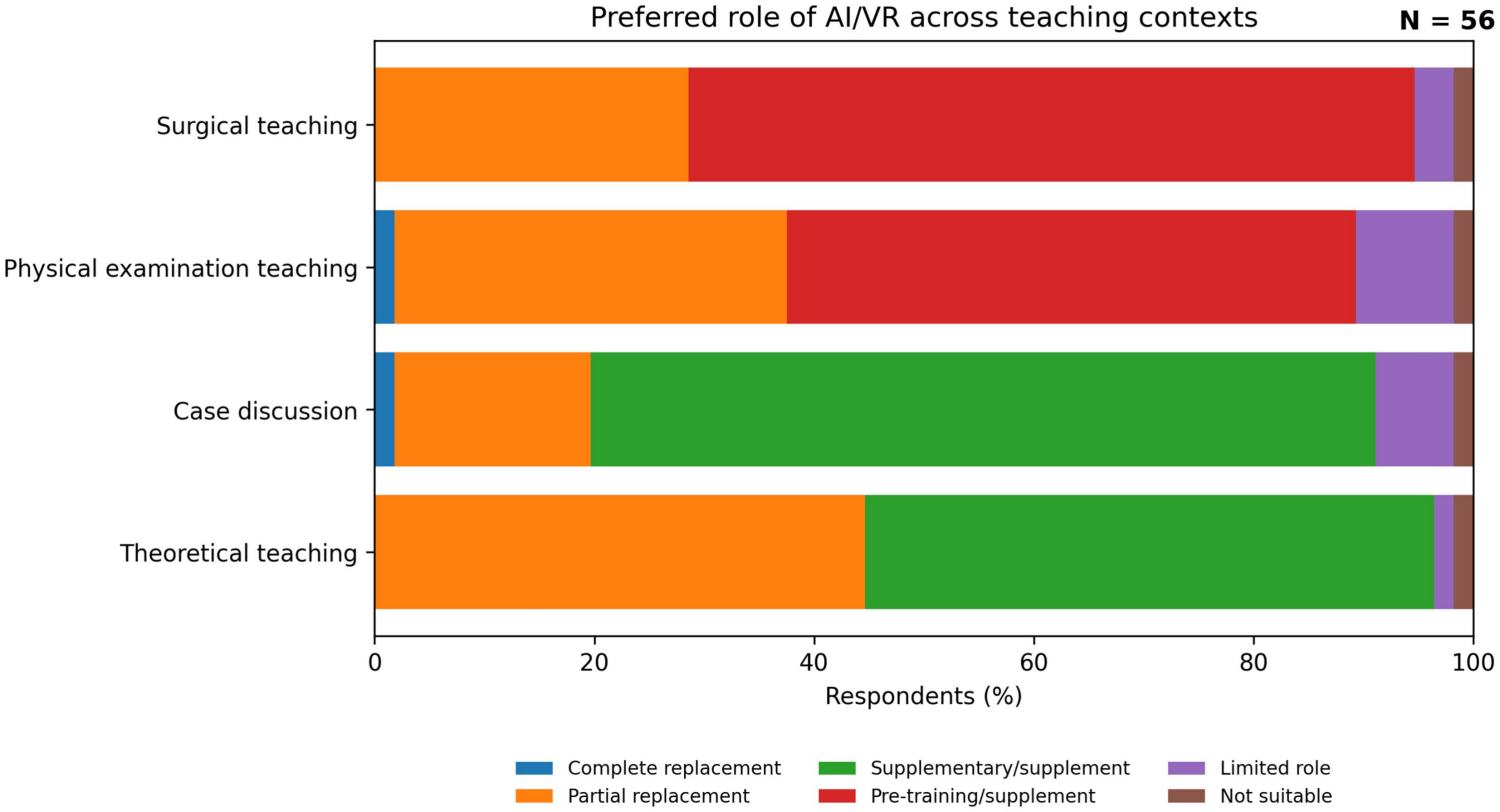
Across teaching contexts, students positioned AI/VR primarily as adjunctive rather than fully substitutive tools.

When asked about the preferred role of AI/VR in specific educational settings, students consistently positioned these technologies as adjunctive rather than fully substitutive tools. In theoretical teaching, 51.8% regarded AI/VR as a supplementary aid and 44.6% considered it suitable for partial replacement of traditional teaching. In case discussion, 71.4% preferred AI as a supplementary discussion tool. In physical examination teaching, 51.8% selected VR as a pre-training tool, whereas in surgical teaching, 66.1% selected VR as a pre-class or preoperative training tool (Figure 2).

**Figure 3:**
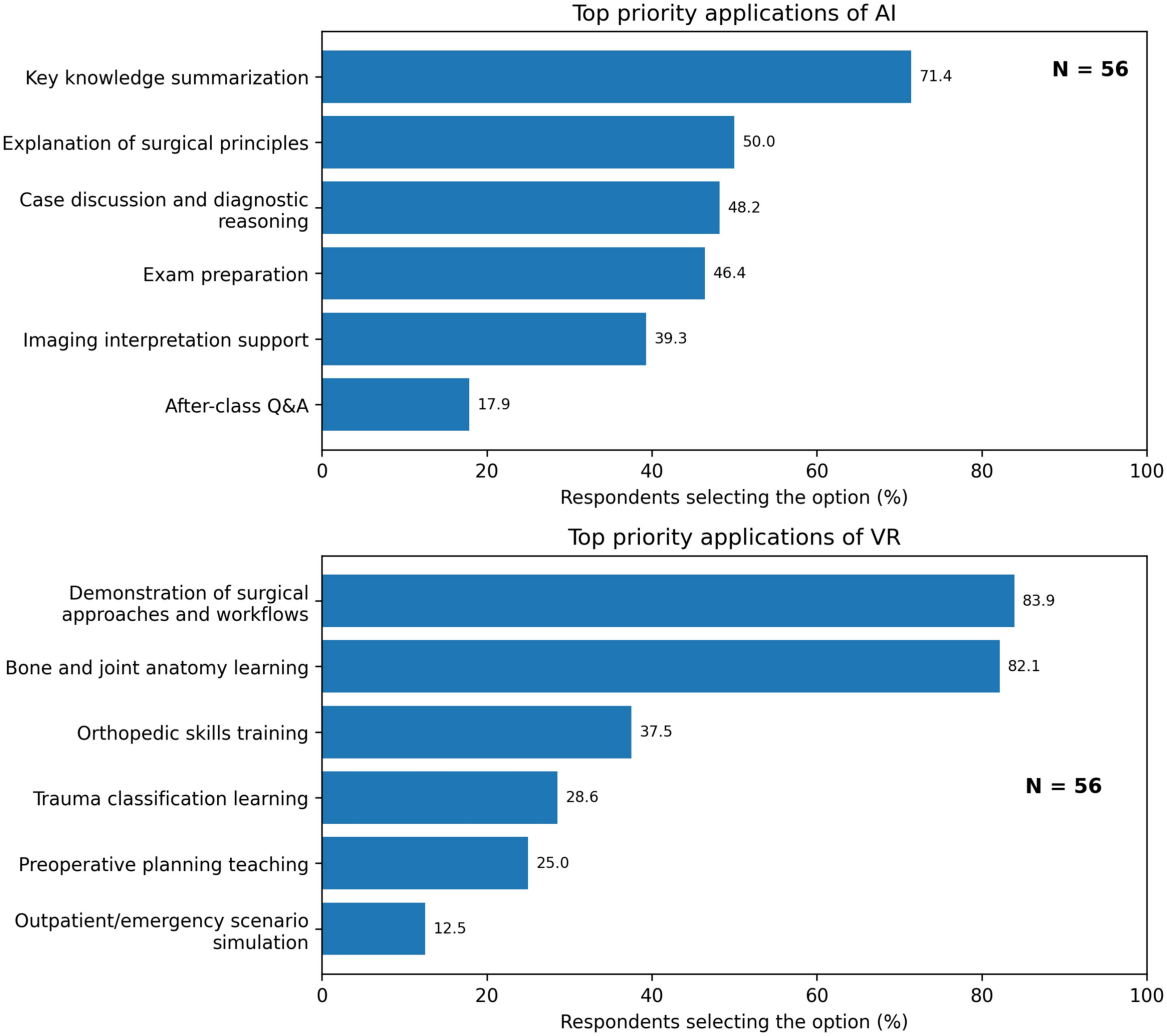
AI and VR were assigned distinct educational functions in orthopedic learning.

Preferences for application scenarios further underscored a division of labor between AI and VR. The most frequently selected AI use cases were key knowledge summarization (71.4%), explanation of surgical principles (50.0%), case discussion and diagnostic reasoning (48.2%), and examination preparation (46.4%). By contrast, VR was prioritized for demonstration of surgical approaches and workflows (83.9%), bone and joint anatomy learning (82.1%), and orthopedic skills training (37.5%) (Figure 3). When asked about a future AI/VR orthopedic teaching platform, the most desired functions were 3D/VR anatomy visualization (85.7%) and surgical simulation (73.2%). Regarding irreplaceable components of orthopedic education, students most often selected real patient encounters in ward/outpatient settings (39.3%) and live surgical observation (30.4%), followed by physical examination demonstration (16.1%).

Distribution of preferred roles for AI/VR in theoretical teaching, case discussion, physical examination teaching, and surgical teaching. For cross-context comparison, response categories were harmonized into broader conceptual groups. Percentages represent the proportion of respondents selecting each role within each teaching context. The figure is descriptive; no inferential test was applied to these categorical role-preference distributions. Percentages are based on all 56 respondents.

Multiple-response results showing the most frequently selected application priorities for AI and VR. Because respondents could select more than one option, percentages do not sum to 100%. AI was primarily preferred for knowledge summarization, reasoning support, and examination preparation, whereas VR was primarily preferred for anatomy learning, surgical workflow visualization, and skills-related pre-training. Results are based on all 56 respondents, who could select multiple options.

### 3.6 Risk perceptions and exploratory subgroup analyses

Risk perception remained substantial despite overall support for implementation. The most strongly endorsed concerns were the need for clear ethical and data-governance standards in AI/VR-assisted orthopedic teaching (4.21 ± 0.65; 87.5% agreement), the possibility that AI may provide inaccurate orthopedic information (4.05 ± 0.64; 85.7% agreement), and the concern that VR simulation may not fully capture the complexity of real clinical settings (4.05 ± 0.70; 78.6% agreement). Concern that AI/VR might weaken authentic teacher-student interaction was comparatively lower (3.29 ± 1.04; 44.6% agreement).

**Figure 4:**
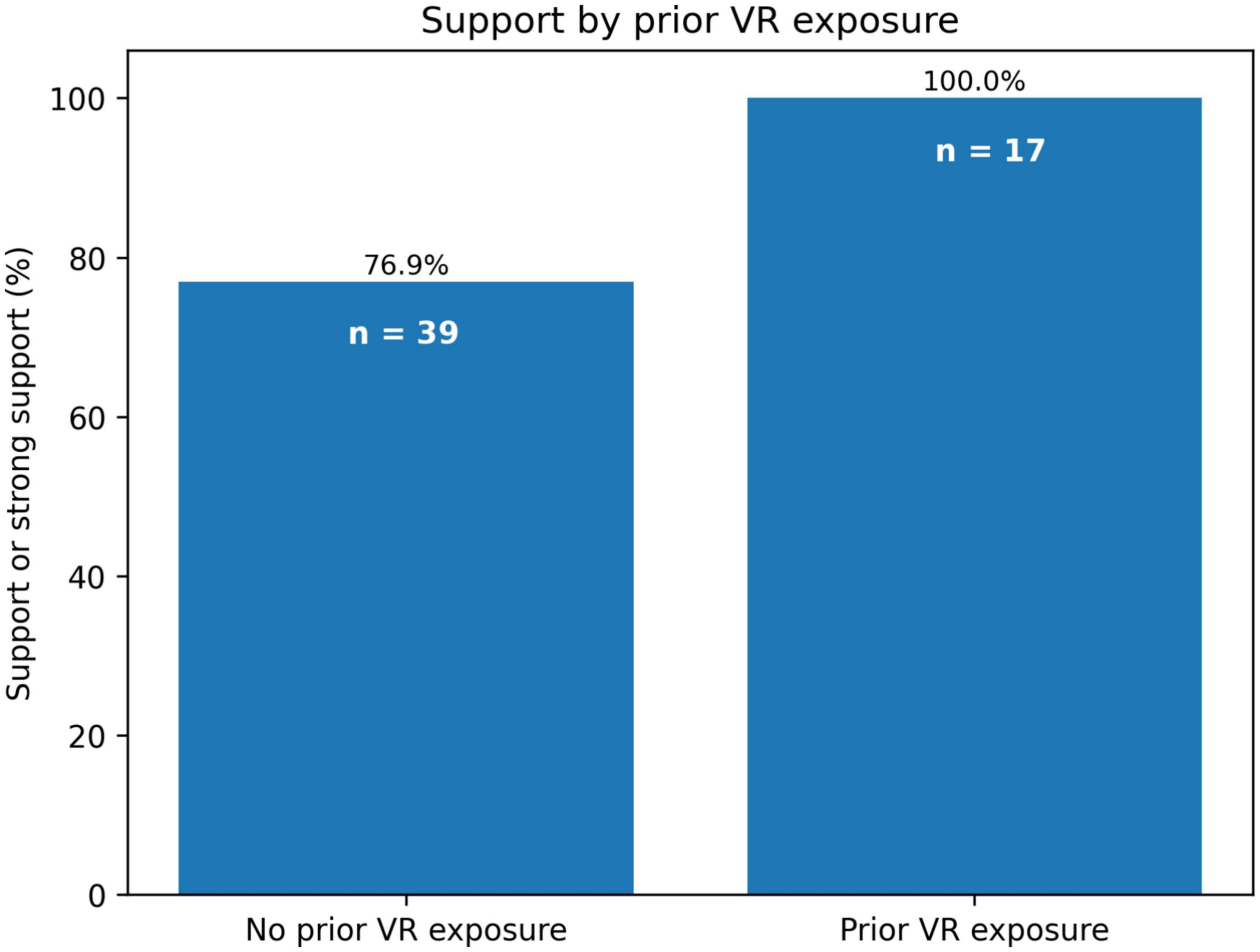
Prior VR/AR/simulation exposure was associated with stronger support for AI/VR integration in orthopedic education.

Exploratory subgroup analyses showed that students with prior VR/AR/simulation exposure reported significantly higher overall support scores than students without such exposure (4.41 ± 0.51 vs. 3.85 ± 0.90, *p* = 0.014; effect size *r* = 0.33). When support was dichotomized, 100.0% of students with prior VR exposure versus 76.9% of those without prior exposure selected ‘support’ or ‘strongly support’ (Fisher’s exact test, *p* = 0.045). Students who had already received systematic orthopedic theory teaching showed slightly lower acceptance scores than those without prior theory exposure (4.14 ± 0.74 vs. 4.43 ± 0.68, *p* = 0.022; effect size *r* = 0.31), whereas senior-stage students (Years 5–8) reported lower risk perception scores than junior-stage students (Year 4) (3.69 ± 0.54 vs. 3.98 ± 0.48, *p* = 0.027; effect size *r* = 0.30). These subgroup findings should be interpreted cautiously because the analyses were exploratory and were not corrected for multiple comparisons; using a conservative Bonferroni adjustment across the three subgroup comparisons, only the association between prior VR exposure and overall support would remain below the 0.05 threshold. In correlation analyses, self-rated AI familiarity was positively associated with acceptance (rho = 0.483, *p* < 0.001), perceived value (rho = 0.518, p < 0.001), educational trust (rho = 0.330, *p* = 0.013), and overall support (rho = 0.305, *p* = 0.022). Self-rated VR familiarity was positively associated with overall support (rho = 0.331, p = 0.013), but not with the other three composite domain scores (Tables 4, 5; Figure 4).

**Table 4:** Exploratory subgroup differences in implementation support, acceptance, and risk perception.

Outcome	Comparison	Group 1, mean ± SD	Group 2, mean ± SD	*p* value (effect size r)
Overall support	Prior VR exposure: Yes vs No	4.41 ± 0.51	3.85 ± 0.90	0.014 (0.33)
Acceptance score	Systematic orthopedic theory teaching: Yes vs No	4.14 ± 0.74	4.43 ± 0.68	0.022 (0.31)
Risk perception score	Senior stage (Years 5–8) vs junior stage (Year 4)	3.69 ± 0.54	3.98 ± 0.48	0.027 (0.30)

Group 1 corresponds to the first group listed in the comparison column. *p* values were derived using Mann–Whitney U tests. Effect size r was calculated as |Z|/sqrt(N); values around 0.10, 0.30, and 0.50 are commonly interpreted as small, moderate, and large effects, respectively.

**Table 5:** Associations of self-rated AI/VR familiarity with acceptance, perceived value, trust, and implementation support.

Predictor	Outcome	Spearman’s Rho	*p* value
AI familiarity	Acceptance score	0.483	<0.001
AI familiarity	Perceived value score	0.518	<0.001
AI familiarity	Educational trust score	0.330	0.013
AI familiarity	Overall support	0.305	0.022
VR familiarity	Acceptance score	0.230	0.088
VR familiarity	Perceived value score	0.225	0.095
VR familiarity	Educational trust score	0.206	0.127
VR familiarity	Overall support	0.331	0.013

Correlations were estimated using Spearman’s rank correlation coefficient.

Proportion of respondents who selected ‘support’ or ‘strongly support’ for further AI/VR integration, stratified by prior exposure to VR/AR/simulation in medical learning. The dichotomized comparison was significant by Fisher’s exact test (*p* = 0.045), although this subgroup analysis was exploratory. Group sizes were *n* = 17 for students with prior VR/AR/simulation exposure and *n* = 39 for those without (total *N* = 56).

## 4 Discussion

Orthopedic education is a particularly informative setting in which to evaluate AI/VR integration because it combines substantial cognitive load with spatial and procedural learning demands. In this context, the key curricular question is not simply whether students welcome new technologies, but how AI and VR should be assigned to different tasks under appropriate supervisory boundaries. In this exploratory cross-sectional survey of clinical-stage students in an eight-year program of clinical medicine, support for AI/VR-assisted orthopedic education was high, yet distinctly bounded. Four principal findings deserve emphasis. First, students showed strong overall acceptance and perceived educational value of AI/VR. Second, they positioned these technologies primarily as adjunctive rather than fully substitutive tools. Third, a clear trust boundary emerged between AI/VR as educational support and AI as a source of recommendations for real patient decision-making. Fourth, students attributed differentiated pedagogical roles to AI and VR: AI was preferentially aligned with summarization, reasoning support, and review, whereas VR was preferentially aligned with anatomy, procedural visualization, and pre-training. Taken together, these findings are consistent with a preliminary, task-specific implementation model conditioned by continued expectations of faculty oversight.

The high level of support observed in this study is consistent with broader literature showing that medical students generally perceive AI as useful for learning support and efficiency, while remaining cautious about accuracy, misuse, and governance (4, 7, 19). Our findings extend this literature by suggesting a specialty-specific implementation logic for orthopedic teaching rather than a generic endorsement of educational technology. Students appeared to value AI/VR where these tools could simplify complexity, improve comprehension, and expand access to preclinical preparation. The positive associations between prior VR exposure, AI familiarity, and implementation support further suggest that structured exposure may facilitate more confident and realistic adoption (8, 12, 15).

At the same time, our data show that acceptance should not be interpreted as endorsement of autonomy. Students expressed willingness to use AI and VR for educational support, but not to extend equivalent trust to AI-generated recommendations for real patient decision-making. This distinction is aligned with contemporary discussions in AI ethics and medical education, in which trust is increasingly understood as contextual and stake-sensitive (20–22). The strong endorsement of teacher review and continued in-person guidance indicates that learners favor supervised deployment rather than pedagogical displacement. In this sense, the present findings support a bounded-use model of AI/VR integration rather than a replacement model.

Another noteworthy finding was the functional differentiation between AI and VR. Students assigned AI to cognitively organized tasks—knowledge summarization, explanation of surgical principles, diagnostic reasoning support, and examination preparation—whereas VR was viewed as particularly valuable for anatomy, fracture classification, operative pathways, and procedural rehearsal. Studies of VR in medical education highlight its strength in immersive, spatially complex, and psychomotor learning environments, while orthopedic studies suggest that VR can improve procedural accuracy, deepen anatomical understanding, and increase learner confidence before real-world exposure (14, 23, 24). By contrast, recent studies of AI and large language models emphasize their utility in information synthesis, personalized tutoring, formative feedback, and simulated dialogue, but also caution against uncritical reliance for factual correctness or clinical judgment (2, 3, 25). Our results therefore support a division-of-labor model in which AI and VR are not treated as interchangeable technologies, but as complementary tools optimized for different instructional tasks within the same curriculum.

The subgroup findings provide further contextual insight, but they should be interpreted as hypothesis-generating. Students with prior VR exposure showed stronger support for implementation, and greater AI familiarity was associated with higher acceptance, perceived value, and support. These patterns may indicate that familiarity promotes more informed and appropriately bounded expectations rather than simple technological enthusiasm. The slightly lower acceptance among students with prior orthopedic theory exposure may reflect more experience-based judgment about the limits of digital tools, whereas the lower risk perception among senior students may indicate increasing clinical maturity. Because subgroup analyses were exploratory and uncorrected for multiplicity, these observations require confirmation in larger samples. These subgroup signals should therefore be regarded as preliminary and hypothesis-generating rather than established associations; under a conservative Bonferroni adjustment across the three comparisons, only the association between prior VR exposure and overall support remained below the 0.05 threshold, and no inferential weight should be placed on these patterns until they are confirmed in larger, adequately powered samples.

The single-center context also matters. PUMC has an intensive eight-year medical curriculum and a tertiary clinical training environment, which may shape students’ exposure to orthopedic teaching, expectations of faculty supervision, and readiness to evaluate new educational technologies. In other institutions, AI/VR adoption may be influenced by resource availability, simulation infrastructure, faculty development, data governance requirements, language context, and local curriculum structure. Therefore, the present findings should not be interpreted as nationally or internationally representative estimates; rather, they provide a context-specific starting point for comparative multi-center work. Accordingly, the present findings should be read as context-specific and exploratory rather than as broadly representative of medical students in general, and any extrapolation to other curricula, countries, or training models should be made cautiously and tested empirically.

These findings suggest a bounded, role-specific curriculum blueprint rather than technology-generic adoption. Current orthopedic teaching in many programs relies heavily on lectures, case discussions, bedside learning, physical examination demonstration, and live operative exposure. A future AI/VR-supported curriculum could preserve those core experiences while adding faculty-reviewed AI summaries before class, AI-supported case reasoning exercises during tutorial work, VR anatomy and fracture-pattern modules before clinical exposure, and VR surgical-pathway rehearsal before observation or skills training. Importantly, neither modality should be positioned as a substitute for bedside teaching, live operative exposure, or supervised clinical reasoning. Faculty remain essential for validation, contextualization, and professional formation.

The next step is therefore not immediate replacement of existing teaching, but staged curriculum development and evaluation. Pilot modules should first test whether AI-supported preparation and VR-based spatial-procedural rehearsal improve knowledge retention, anatomy understanding, procedural confidence, OSCE performance, or transfer to supervised clinical tasks. Longitudinal or interventional studies should then compare AI/VR-supported curricula with existing teaching pathways. Future work should also extend beyond undergraduate students to early trainees, residents, and practicing physicians, whose clinical responsibilities and tolerance for automation risk differ substantially. For developers of AI/VR educational technologies, our findings imply the need for faculty review interfaces, transparent content provenance, embedded assessment and feedback, privacy safeguards, and design features that support critical reasoning rather than passive reliance.

This study has several strengths. It examined AI and VR together rather than in isolation, focused on a specialty in which both technologies are pedagogically salient, and used a multidimensional questionnaire that moved beyond acceptance alone to include perceived value, trust boundaries, substitution boundaries, application priorities, and risk perceptions. These features allowed us to identify a more nuanced pattern than simple technological optimism or pessimism. Nonetheless, several limitations should be acknowledged. First, the study was exploratory and single-center, with a relatively small sample size and a modest response proportion (56 of approximately 300 invited students, 18.7%). No *a priori* power calculation was performed because we attempted to invite the full eligible cohort rather than test a predefined effect size. The results therefore should be interpreted as exploratory and hypothesis-generating, not definitive estimates of student attitudes.

Second, non-response bias and self-selection bias are possible. Students with stronger interest in digital learning or greater availability may have been more likely to respond, whereas students who were less engaged or more skeptical may be underrepresented. This limits representativeness and statistical power, particularly for subgroup analyses. Third, the questionnaire was newly developed for this study. Although the author team reviewed items for face and content relevance and internal consistency was acceptable to excellent across domains, formal Delphi validation, pilot psychometric testing, construct validation, and exploratory factor analysis were not performed. Fourth, the study measured perceptions rather than educational effectiveness, learning outcomes, behavioral adoption, or patient-related consequences. Finally, the institutional and national context may limit generalizability to other curricula, healthcare systems, and technological environments. Because more digitally enthusiastic students were plausibly overrepresented, the observed levels of acceptance, perceived value, and overall support may be inflated relative to the full eligible cohort; the reported estimates should therefore be regarded as upper-bound rather than representative figures. This further reinforces the need to interpret the findings as exploratory and to confirm them in larger, more representative samples.

## 5 Conclusion

In conclusion, students in this 8-year medical program broadly supported integrating AI and VR into orthopedic education, but in a selective and bounded manner. Their responses suggest a preliminary role-specific model in which AI primarily enhances knowledge organization and reasoning support, whereas VR primarily supports spatial-procedural understanding and pre-training, with faculty oversight remaining indispensable. For orthopedic educators, the key implication is not whether to adopt AI/VR in the abstract, but how to test and integrate them in an ethically governed, educationally targeted, and outcome-evaluated manner.

## Data Availability

The raw data supporting the conclusions of this article will be made available by the authors, without undue reservation.
