# Supplementary material for: Beyond acceptance: bounded use of AI and VR in orthopedic education
Source: Front Med (Lausanne). 2026 Jul 15;13:1856528. doi: 10.3389/fmed.2026.1856528 (PMC13416917; doi:10.3389/fmed.2026.1856528)
Supplement: Supplementary file 1 [file Table_1.docx]

**Appendix 1. The Questionnaire used to conduct the study (In English)**

**English translation of the questionnaire administered in Chinese via Wenjuanxing**

**Title of the questionnaire**
Acceptance, Trust Boundaries, and Application Preferences for AI/VR-Assisted Orthopedic Education Among Students in the 8-Year Medical Program at XX Medical College

**Instructions to participants**
Dear student,
Hello. This questionnaire aims to understand the views and needs of students in the 8-year medical program at XX Medical College regarding the use of artificial intelligence (AI) and virtual reality (VR) in orthopedic education. The questionnaire is anonymous and will be used for research purposes only. No personally sensitive information is involved. Please answer all questions according to your true opinions. The questionnaire takes approximately 5–8 minutes to complete. Thank you for your support.

**Section 1. Basic information**

**Q1. What is your sex?**
Single choice

- Male
- Female
- Prefer not to say

**Q2. What is your current training stage?**
Single choice

- Year 4
- Years 5–6
- Years 7–8

**Q3. Have you already received systematic orthopedic theory teaching?**
Single choice

- Yes
- No

**Q4. Have you participated in orthopedic observation, internship, or rotation?**
Single choice

- Yes
- No

**Q5. How many times have you observed orthopedic surgery?**
Single choice

- Never
- 1–2 times
- 3–5 times
- More than 5 times

**Q6. Are you currently considering a future specialty related to surgery?**
Single choice

- Yes
- No
- Uncertain

**Q7. Are you currently considering a future specialty related to orthopedics?**
Single choice

- Yes
- No
- Uncertain

**Section 2. Previous exposure to AI/VR**

**Q8. Which of the following AI tools have you used to assist medical learning?**
Multiple choice

- ChatGPT / Gemini / Claude
- DeepSeek / Doubao / Qianwen / Yuanbao / ERNIE Bot / Tongyi / iFlytek Spark or other large language model tools
- AI-based question banks or learning assistant tools
- AI tools for medical image analysis
- Other AI tools
- I have never used AI tools for learning

**Q9. How often do you use AI tools to assist medical learning?**
Single choice

- Never
- Rarely
- Occasionally
- Often
- Almost daily

**Q10. Have you ever used VR/AR/simulation systems for medical learning?**
Single choice

- Yes
- No

**Q11. If yes, in which scenarios have you mainly used VR/AR/simulation systems?**
Multiple choice

- Anatomy learning
- Surgical simulation
- Clinical skills training
- Case simulation
- Teaching demonstration / science communication
- Other
- No prior exposure

**Q12. How familiar are you with AI technology?**
5-point scale
1 = Not familiar at all
5 = Very familiar

**Q13. How familiar are you with VR technology?**
5-point scale
1 = Not familiar at all
5 = Very familiar

**Section 3. Overall acceptance of AI/VR-assisted orthopedic education**

For the following items, please indicate your level of agreement.
5-point Likert scale
1 = Strongly disagree
2 = Disagree
3 = Neutral
4 = Agree
5 = Strongly agree

**Q14. I support the introduction of AI-assisted learning tools into orthopedic education.**
**Q15. I support the introduction of VR/immersive simulation training into orthopedic education.**
**Q16. AI/VR can increase my interest in learning orthopedic content.**
**Q17. AI/VR can improve the intuitiveness and comprehensibility of orthopedic teaching.**
**Q18. AI/VR can serve as a useful supplement to traditional orthopedic teaching.**
**Q19. If such resources are provided, I would be willing to actively use AI/VR for orthopedic learning.**
**Q20. I think AI/VR is more suitable for preview and post-class review in orthopedic teaching.**
**Q21. If AI/VR could provide immediate feedback, I would be more willing to study orthopedic content after class.**

**Section 4. Perceived value**

For the following items, please indicate your level of agreement.
5-point Likert scale
1 = Strongly disagree
2 = Disagree
3 = Neutral
4 = Agree
5 = Strongly agree

**Q22. AI helps me understand basic orthopedic knowledge and concepts.**
**Q23. AI helps me organize diagnostic and therapeutic reasoning in orthopedic cases.**
**Q24. AI helps me summarize key points and prepare for examinations in orthopedic courses.**
**Q25. VR helps me understand bones, joints, and spatial anatomy.**
**Q26. VR helps me understand fracture classification, internal fixation, and surgical pathways.**
**Q27. VR helps me engage in pre-training before formal clinical teaching.**
**Q28. Compared with text or PowerPoint alone, AI/VR helps me better understand complex orthopedic content.**
**Q29. AI/VR helps improve my efficiency in learning orthopedics.**

**Section 5. Trust boundaries**

For the following items, please indicate your level of agreement.
5-point Likert scale
1 = Strongly disagree
2 = Disagree
3 = Neutral
4 = Agree
5 = Strongly agree

**Q30. I trust AI explanations of basic orthopedic knowledge.**
**Q31. I trust the value of AI in supporting the learning of typical orthopedic cases.**
**Q32. I trust the value of VR in orthopedic spatial anatomy teaching.**
**Q33. I trust the value of VR in demonstrating orthopedic procedural workflows.**
**Q34. I trust AI-generated orthopedic clinical recommendations sufficiently for real patient decision-making.**
**Q35. I believe AI/VR educational content should be formally used only after teacher review.**
**Q36. Even if AI/VR performs well, I still believe orthopedic teaching cannot do without in-person teacher guidance.**
**Q37. AI/VR can replace a considerable part of traditional orthopedic teaching.**

**Section 6. Substitution boundaries and application preferences**

**Q38. In orthopedic theoretical teaching, what role do you think AI/VR should play?**
Single choice

- Completely replace traditional teaching
- Partially replace traditional teaching
- Serve as a supplementary aid
- Have limited usefulness
- Not suitable for use

**Q39. In orthopedic case discussion, what role do you think AI should play?**
Single choice

- Completely replace teacher explanation
- Partially replace teacher explanation
- Serve as a supplementary discussion tool
- Have limited usefulness
- Not suitable for use

**Q40. In orthopedic physical examination teaching, what role do you think VR should play?**
Single choice

- Completely replace live demonstration on real persons
- Partially replace live demonstration on real persons
- Serve as a pre-training tool
- Have limited usefulness
- Not suitable for use

**Q41. In orthopedic surgical teaching, what role do you think VR should play?**
Single choice

- Completely replace live surgical observation
- Partially replace live surgical observation
- Serve as a preoperative / pre-class training tool
- Have limited usefulness
- Not suitable for use

**Q42. In your opinion, in which orthopedic teaching scenarios should AI be prioritized?**
Multiple choice, up to 3 options

- Key knowledge summarization
- Case discussion and diagnostic/therapeutic reasoning
- Imaging interpretation support
- Explanation of surgical principles
- After-class Q&A
- Examination preparation
- OSCE/case-station simulation
- Other

**Q43. In your opinion, in which orthopedic teaching scenarios should VR be prioritized?**
Multiple choice, up to 3 options

- Bone and joint anatomy learning
- Understanding of trauma/fracture classification
- Demonstration of surgical approaches and workflows
- Orthopedic skills training
- Outpatient/emergency scenario simulation
- Teamwork training
- Preoperative planning teaching
- Other

**Q44. If the school develops an AI/VR orthopedic teaching platform in the future, which functions would you most like it to include?**
Multiple choice, up to 3 options

- Intelligent Q&A
- Interactive case simulation
- Personalized learning-path recommendations
- 3D/VR anatomical visualization
- Surgical simulation
- Automated feedback and error analysis
- Teacher feedback interface
- Other

**Section 7. Risk perception**

For the following items, please indicate your level of agreement.
5-point Likert scale
1 = Strongly disagree
2 = Disagree
3 = Neutral
4 = Agree
5 = Strongly agree

**Q45. I am concerned that AI may provide incorrect or inaccurate orthopedic medical information.**
**Q46. I am concerned that students may become overly dependent on AI and weaken their independent thinking.**
**Q47. I am concerned that VR simulation cannot fully reflect the complexity of real clinical environments.**
**Q48. I am concerned that AI/VR may weaken authentic teacher–student interaction.**
**Q49. I believe that the use of AI/VR in orthopedic teaching requires clear ethical and data-governance standards.**
**Q50. I am concerned that AI/VR may lead students to overestimate their actual clinical competence.**

**Section 8. Overall attitude and open-ended item**

**Q51. Overall, do you support the further introduction of AI/VR into orthopedic teaching in the 8-year medical program at XX Medical College?**
Single choice

- Strongly do not support
- Do not support
- Neutral
- Support
- Strongly support

**Q52. In your opinion, which component of orthopedic teaching is least replaceable by AI/VR?**
Single choice

- Teacher lecturing
- Demonstration of clinical physical examination
- Real patient encounters in wards/outpatient settings
- Live surgical observation
- Clinical reasoning training
- Other
